# The Role of Placental Tryptophan Catabolism

**DOI:** 10.3389/fimmu.2014.00230

**Published:** 2014-05-19

**Authors:** Peter Sedlmayr, Astrid Blaschitz, Roland Stocker

**Affiliations:** ^1^Institute of Cell Biology, Histology and Embryology, Medical University of Graz, Graz, Austria; ^2^Victor Chang Cardiac Research Institute, Darlinghurst, NSW, Australia

**Keywords:** pregnancy, placenta, intrauterine growth restriction, fetal growth restriction, preeclampsia, vasotonus, feto-maternal tolerance, immunoregulation

## Abstract

This review discusses the mechanisms and consequences of degradation of tryptophan (Trp) in the placenta, focusing mainly on the role of indoleamine 2,3-dioxygenase-1 (IDO1), one of three enzymes catalyzing the first step of the kynurenine pathway of Trp degradation. IDO1 has been implicated in regulation of feto-maternal tolerance in the mouse. Local depletion of Trp and/or the presence of metabolites of the kynurenine pathway mediate immunoregulation and exert antimicrobial functions. In addition to the decidual glandular epithelium, IDO1 is localized in the vascular endothelium of the villous chorion and also in the endothelium of spiral arteries of the decidua. Possible consequences of IDO1-mediated catabolism of Trp in the endothelium encompass antimicrobial activity and immunosuppression, as well as relaxation of the placental vasotonus, thereby contributing to placental perfusion and growth of both placenta and fetus. It remains to be evaluated whether other enzymes mediating Trp oxidation, such as indoleamine 2,3-dioxygenase-2, Trp 2,3-dioxygenase, and Trp hydroxylase-1 are of relevance to the biology of the placenta.

## Introduction

**Figure 1:**
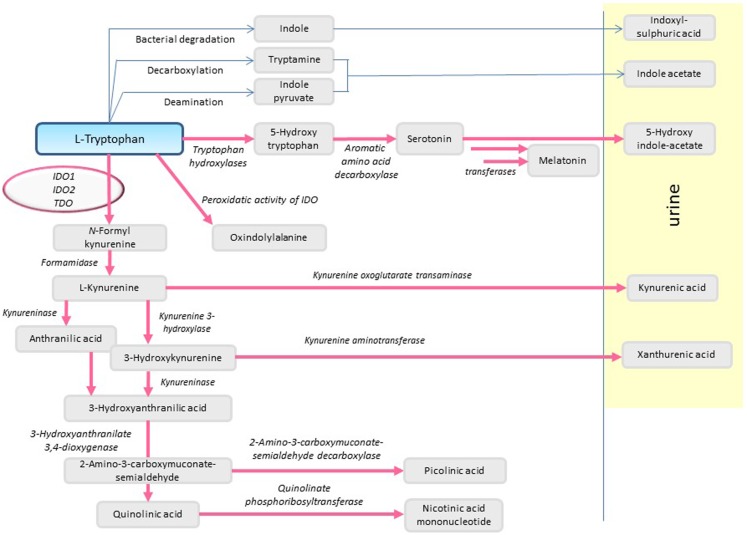
**Pathways of Trp degradation**.

l-Tryptophan (l-Trp) is a hydrophobic amino acid with a chemical structure based on an indole ring. l-Trp is the least abundant essential amino acid, and therefore needs to be supplied by nutrients such as meat, fish, milk, eggs, vegetables, nuts, and seeds such as soybeans, sesame, and sunflower seeds. The daily requirement of adults is in the range of 3 mg/kg (1). Apart from protein synthesis, l-Trp is utilized for the synthesis of the neurotransmitter serotonin and the hormone melatonin in the pineal gland. Degradation of Trp in mammals occurs predominantly (>95%) along the kynurenine pathway, leading to synthesis of nicotinamide adenine dinucleotide (NAD^+^) (2) (Figure 1).

The first step in the oxidative metabolism of l-Trp along the kynurenine pathway is catalyzed independently by three different enzymes: indoleamine 2,3-dioxygenase-1 (IDO1), indoleamine 2,3-dioxygenase-2 (IDO2), and Trp 2,3-dioxygenase (TDO). By incorporating molecular oxygen, these enzymes convert l-Trp to *N*-formyl-kynurenine, which is then converted to kynurenine. l-Trp degradation not only leads to depletion of the amino acid but also to the production of metabolites displaying various biological activities.

## Tryptophan-Degrading Enzymes

### Indoleamine 2,3-dioxygenase-1

Indoleamine 2,3-dioxygenase-1 (IDO, indoleamine-pyrrole 2,3-dioxygenase), reviewed in Ref. (3), is a cytosolic heme-containing enzyme sharing some sequence similarity with myoglobin (4). IDO1 has been conserved through 600 million years of evolution (5). The protein is encoded by the IDO1 (also INDO) gene that is located on chromosome 8, contains 10 exons, and a promoter region that includes 2 interferon (IFN) – stimulated responsive elements. Human IDO cDNA encodes a protein of 403 amino acids with molecular weight of about 45 kDa (6, 7). The primary sequence of human IDO1 shows 57 and 58% identity to mouse and rat IDO1, respectively, whereas no sequence homology was found to rat TDO (8). IDO1 requires activation by reduction of its Fe^3+^-heme form. Early studies suggested that superoxide anion is responsible for this reductive activation (9), although more recent studies indicate formation of Fe^2+^-IDO1 is accomplished by cytochrome *b_5_* plus cytochrome P450 reductase and NADPH (10). Despite numerous studies, the mechanism by which IDO1 oxidizes l-Trp to *N*-formyl-kynurenine remains controversial, with both concerted incorporation of the two oxygen atoms and consecutive insertions of single oxygen atoms into the substrate being proposed (11). Fe^2+^-IDO1 rapidly autoxidizes to the inactive Fe^3+^-IDO1 (12). In the presence of hydrogen peroxide (H_2_O_2_), IDO1 takes on a peroxidase activity that can lead to the oxidation of l-Trp to oxyindolylalanine, and protein oxidation leading to IDO1 inactivation (13). IDO1 prefers l-Trp as a substrate but may also cleave d-Trp and other indoleamines such as tryptamine. In contrast to rabbit IDO, however, the human enzyme does not act on serotonin (14). 1-Methyltryptophan (1-MT) is a compound commonly used to inhibit IDO1 activity, although it is now recognized that the enzyme is also capable of metabolizing 1-MT. The l-isoform of 1-MT has been reported to be a more efficient inhibitor of IDO1 than the d-isomer (15, 16). Further IDO inhibitors are discussed in (17, 18). INCB024360 and Amg-1 have been reported to block IDO1 selectively, with no effect on IDO2 and TDO (19, 20).

In humans, high Trp-degrading activity has been described in the lung, the intestine, and particularly in the term placenta, where it was attributed to IDO1 (21). At that time, however, a possible contributory role of extrahepatic TDO and/or IDO2 was not envisaged. IDO1 is also detected in the mammalian epididymis, where its absence generates an inflammatory state and correlates with an increase in abnormal spermatozoa in IDO1 gene knockout (IDO1^−/−^) mice (22). On a cellular basis, constitutive expression of IDO1 has been found in subsets of dendritic cells (DC) (23), including DC of tumor-draining lymph nodes (24). Moreover, IDO1 has been reported in eosinophils (25), in glandular and surface epithelium of the endometrium and Fallopian tubes (26), and in placental endothelial cells (26–28). IDO1 is also present in microvascular endothelial cells of tumors (29) (Blaschitz, unpublished observations for hepatocellular carcinoma) and the heart in human septic shock (30). Regulatory T cells have been reported to induce the expression of IDO1 in vascular endothelial cells of transplanted hearts in rats (31). Diverging inducibility of IDO1 has been reported for different types of normal endothelial cells, as summarized in Table 1.

**Table 1:** **Expression of IDO1 in various types of vascular endothelium**.

Constitutive	Following inflammation *in vivo*	Following cytokine stimulation (IFNγ and/or TNF-α or IL-1β)	Constitutively negative expression following cytokine stimulation not tested	No or little even after stimulation with IFNγ
Chorionic vascular endothelium (26, 28, 59)	Mouse brain vascular endothelium (63, 125)	HUVEC (28, 116)	Iliac vein endothelial cells (28)	HSVEC (IDO upregulated after mycoplasma infection) (116)
Arteries and capillaries of the decidua (26, 28)	Mouse microvascular endothelium in kidney and intestine during cerebral malaria infection or after administration of LPS (63)	HAEC (10, 28)		RAEC (116)
Pulmonary capillaries (Blaschitz, unpublished observations), expression enhanced in hypoxia (62)	Human microvascular endothelial cells in heart and kidney in septic shock (30)	HBMEC (126)		IMAEC (116)
		Vascular endothelial cells following incubation of porcine, rabbit, rat, and mouse coronary, carotid, and aortic arteries with IFN-γ (63)		

*HUVEC, human umbilical vein endothelial cells; HAEC, human aortic endothelium cells; HSVEC, human saphenous vein endothelial cells; RAEC, radial artery endothelial cells; IMAEC, internal mammary artery endothelial cells; HBMEC, human brain microvascular endothelial cells*.

Indoleamine 2,3-dioxygenase-1 can be induced by IFN-γ acting via Janus kinase (JAK)/signal transducer and activator of transcription (STAT) signaling, type I interferons, prostaglandin E2, lipopolysaccharide (LPS), DNA regions containing a high frequency of cytosine nucleotides adjacent to guanine nucleotides (CpG islands), and other factors in a variety of cell types such as DC, macrophages, epithelial and endothelial cells, Langerhans cells, astrocytes, and T lymphocytes. Also hormones such as estrogen (32) and human chorionic gonadotropin (hCG) (33–35) induce IDO1 expression. Upregulation of IDO1 in DC by hCG is independent of IFN-γ (34). The compounds which induce IDO1 expression in DC have been reviewed previously (36). In addition to IDO1 induction, blockade of cyclooxygenase (COX)-2 has been reported to downregulate IDO1 expression in tumors of animal models, suggesting an interplay between these two enzymes (37).

### Indoleamine 2,3-dioxygenase-2

Indoleamine 2,3-dioxygenase-2 (IDO-like protein, INDOL1, proto-IDO) was described first in 2007 (38, 39) and has been reviewed recently (40). IDO2 has a molecular weight of 47 kDa, is composed of 420 amino acid residues, and displays 43% identity with IDO1 at the amino acid level. The gene for IDO2 is located on chromosome 8, adjacent to its paralog IDO1, and may have arisen from gene duplication (41). Alternatively spliced transcripts have been described (42), however, it is unclear whether they are all translated into protein. Two genetic polymorphisms in the human gene encoding IDO2 ablate its enzymatic activity, such that about 50% of Caucasians and Asians and 25% of Africans lack functional IDO2 alleles (42).

Expression of IDO2 mRNA has been described in kidney, liver, epididymis, testis, uterus, placenta, and brain (15, 38, 43). IDO2 has also been found in sperm tails (38), pancreatic cancer cell lines (44), and tumors of the stomach, colon, and kidney (45). Similar to IDO1, IFN-γ upregulates IDO2 expression in DC (45), mesenchymal stem cells, macrophages, and astrocytes (43), although IFN-γ does not necessarily induce IDO1 and IDO2 simultaneously (19, 43). Preferential inhibition of IDO2 by a particular 1-MT enantiomer is contentious. An early report of more efficient inhibition by the d-isomer of 1-MT (42) has not been confirmed (16, 46) [for discussion see (40)]. Tenatoprazole has been reported to inhibit IDO2 without affecting IDO1 or TDO, although this compound also displays other biological effects (47).

### Further Trp-degrading enzymes

Like IDO1, TDO is a cytosolic heme dioxygenase. It is coded for by the TDO2 gene and displays only 10% amino acid sequence identity with IDO1 (48). The structure and function of TDO and IDO1 have been compared previously (49). TDO is a homotetramer with a subunit molecular weight of 103 kDa. In contrast to IDO1, TDO is enantiomer-specific and only cleaves the l-isoform of Trp (48). Although thought initially to be expressed in the liver only, TDO is also present in placenta (50), brain (51), and a variety of human carcinomas. In the mouse endometrium, TDO is induced at the time of implantation (52). The expression of TDO is upregulated by glucocorticoids (53, 54) and by l-Trp (55). 1-MT does not inhibit TDO, while the compound 680C91 has been reported to selectively block TDO but not IDO1 (56).

Tryptophan hydroxylases (Tph-1 and Tph-2) convert Trp to 5-hydroxytryptophan for subsequent synthesis of serotonin and melatonin, rather than being involved in the kynurenine pathway. Tph-1 and Tph-2 are homologous enzymes with 71% amino acid sequence identity, and with their respective genes located on chromosomes 11 and 12. Mast cells are the major source of Tph-1, whereas Tph-2 is expressed predominantly in neuronal cells of the brain stem (57).

## Placental Expression and Localization of Trp-Degrading Enzymes

There are several, albeit partly conflicting reports on the localization of IDO1 in the human placenta.

### IDO1 in the chorionic vascular endothelium

**Figure 2:**
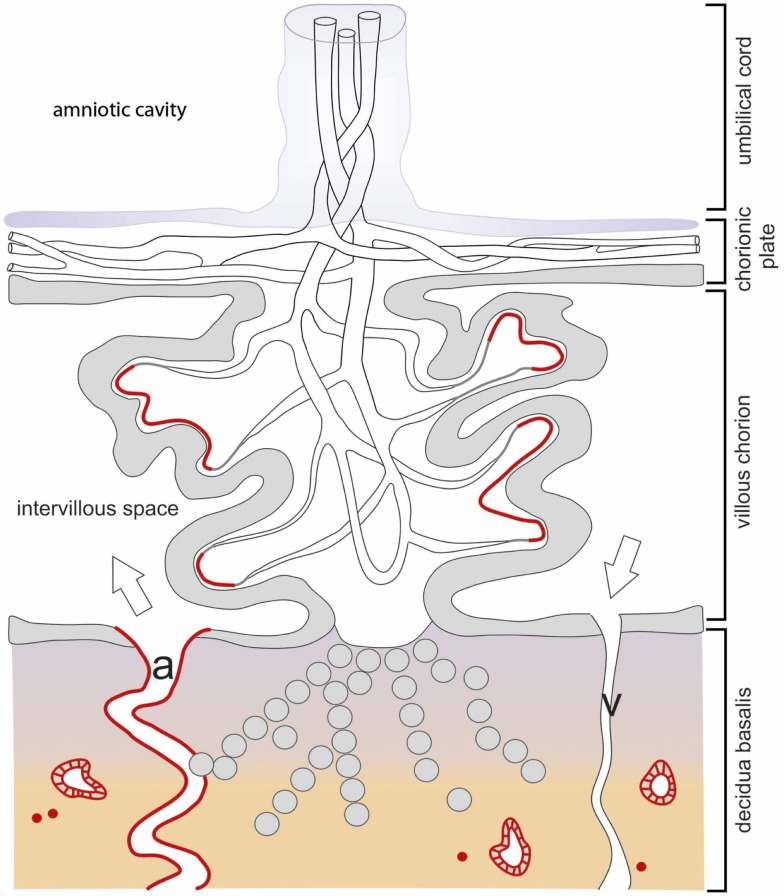
**Schematic drawing of the localization of IDO1 in the human placenta during first trimester pregnancy**. The chorionic villus is the structural element involved in feto-maternal exchanges. The stem villi originate from the chorionic plate and ramify into villous branches. They consist of a core of mesenchymal connective tissue containing vessels, which are in contact with the fetal vasculature via the umbilical cord. The chorionic villi are covered by a double layer of villous trophoblast (the upper syncytiotrophoblast and the lower cytotrophoblast) separating the fetal closed blood circulation from the intervillous space, which is filled with maternal blood which is supplied via the uterine spiral arteries (a) and discharged via the uterine veins (v). Some of the villi are anchored into the maternal *decidua basalis* by roots built of extra-villous cytotrophoblast cells, which also invade the maternal *decidua*. The IDO1 expression sites are highlighted in red color and refer to the villous subtrophoblastic capillaries, few immune cells of the *decidua*, and the epithelium of uterine glands.

**Figure 3:**
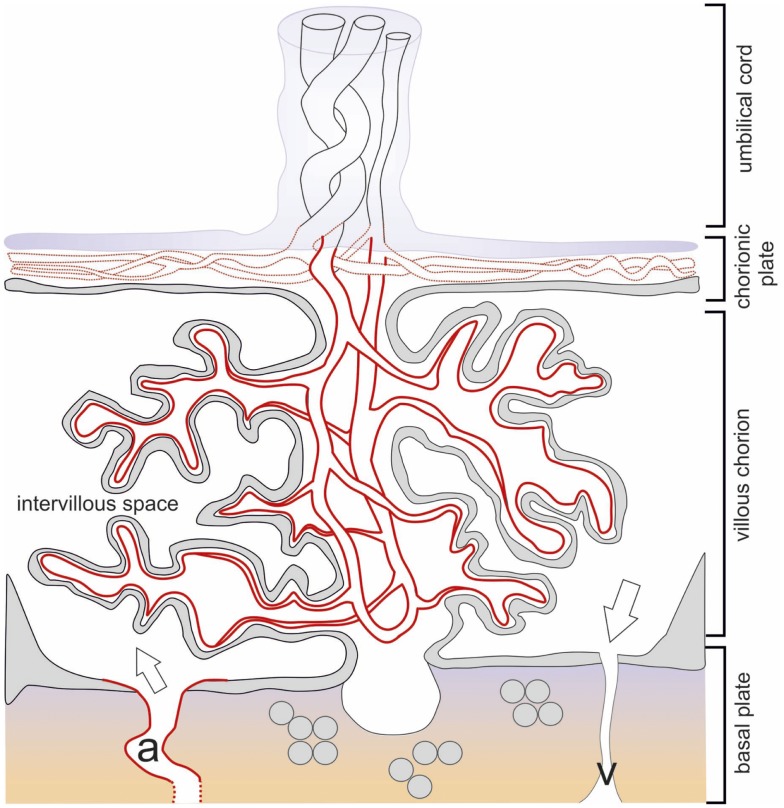
**Schematic drawing of the term placenta with the basal plate after delivery**. The structures of placental architecture are described in the legend to Figure 1. Here, the branching of the villous tree has increased, the villous trophoblast is largely reduced to the syncytiotrophoblast. IDO1 protein is indicated by red color broken red lines indicate partial expression. All endothelia of the vessels of the villous chorion express IDO1, while only part of the vessels of the chorionic plate and none of the umbilical cord vessels are positive. Openings of maternal arteries (a) express IDO1 whereas veins do not.

In early pregnancy, IDO1 expression is restricted exclusively to immediately subtrophoblastic capillaries (Figure 2), and it increases with advancing gestational age. In term placenta, the endothelium of larger vessels in stem villi and some arteries and veins of the chorionic plate stain positive for IDO1 protein, whereas the vessels of the umbilical cord remain IDO1 negative (28, 58, 59) (Figure 3). Similar results for chorionic vascular endothelial expression of IDO1 have been described in rhesus monkeys and common marmosets (60). This increase in protein expression correlates with both the amount of mRNA in the placenta and the increase in the placental kynurenine-to-Trp ratio, a surrogate measure of IDO activity. In term placentas at delivery, the kynurenine-to-Trp ratio measured in the blood obtained from vessels of the chorionic plate is far higher than that in the peripheral blood of healthy blood donors (28). This suggests that endothelial IDO1 within placental vessels is highly active beyond the cessation of placental blood circulation at delivery. Consistent with this, endothelial cells isolated from the chorionic plate of term placenta express IDO1 mRNA, in contrast to endothelial cells isolated from human umbilical vein, iliac vein, or aorta (28). Moreover, expression of the aryl hydrocarbon receptor (AhR) AhR, a receptor for kynurenine, has been reported for syncytiotrophoblasts, the endothelium of large vessels in the chorionic villi, and in the endothelium of umbilical cord arteries and veins (61).

### IDO1 in vascular endothelium of the decidua and the uterus

In endometrium of non-pregnant women, vascular endothelium does not express IDO1 protein, whereas the protein is expressed in HLA-DR-negative endothelium of spiral arteries and in capillaries. In contrast, the HLA-DR-positive endothelium of veins of the decidua is negative for IDO1 as assessed by immunohistochemistry (Figure 2). During mid-gestation, endothelial expression IDO1 extends to the inner but not the outer layer of the myometrium (26, 28). Thus, endothelial IDO1 is increasingly expressed the tissue closer to the feto-maternal interface, similar to the situation in the chorion. It is noticeable that constitutive expression of IDO1 in vascular endothelium is limited to the placenta, the uterus, and the lungs (28, 62) (Blaschitz, unpublished observations). In contrast, IDO1 appears to be more generally expressed in the endothelium under conditions of systemic inflammation (63).

### IDO1 in epithelium of the endometrium and the decidua

Expression of IDO1 increases over the course of the menstrual cycle in the surface and glandular epithelium of the endometrium, just as the protein is expressed in cervical glands and epithelium of Fallopian tubes in non-pregnant women. Cervical mucus displays some Trp-degrading activity (26). In first trimester decidua, IDO1 is present in glandular epithelial cells (26, 59).

### IDO1 in the trophoblast

There is discrepancy among publications as to whether IDO1 is expressed in trophoblast cells. Earlier publications reported IDO1 to be present in first trimester (59) and/or term placenta syncytiotrophoblast (26, 58, 64) and in extra-villous cytotrophoblast cells (58, 64). Hönig et al. described IDO1 in the invasive extra-villous trophoblast in the *decidua basalis* and trophoblast giant cells (58). These observations were challenged in a subsequent publication that also discussed possible reasons for the apparent discrepancies (28). In keeping with this, Wang et al. (65) reported that isolated first trimester trophoblast cells do not constitutively express IDO1 mRNA and protein. However, treatment with polyinosinic–polycytidylic acid [poly(I:C)] (a synthetic double-stranded RNA, which mimics viral RNA and is a ligand of the Toll-like receptor-3) induced IDO1 mRNA and Trp-degrading activity in the trophoblasts (65). Conditioned media from poly(I:C)-treated trophoblast cells suppresses T cell DNA synthesis, and IFN-β was identified as the mediator of this effect via the induction of IDO1 (65). In human placental explants, IDO1 mRNA was found after 24 h of culture, the expression increased following LPS stimulation (66).

Recently, expression of IDO1 mRNA was described in cultured third trimester human placental cytotrophoblast cells, with higher expression in male than in female CT cells (67). However, these cytotrophoblast preparations also contained CD34 mRNA (Cvitic and Desoye, personal communication), so that contamination with endothelial cells cannot be excluded. Contaminating endothelial cells may also explain similar findings reported earlier by Dong et al. (68). In mice, placental IDO1 expression was found to be limited to trophoblast giant cells (69).

### IDO1 in other placental cell types

Indoleamine 2,3-dioxygenase-1 expression has been reported in macrophages of the villous stroma (59, 64). However, this finding was contested subsequently by the observation that IDO1-positive chorionic cells consistently co-expressed CD34 (28), suggesting that in the villous stroma IDO1 is restricted to endothelial cells. IDO1 protein is absent from the majority of macrophages and DC in the decidua (70, 71). However, IDO1 can be induced in these cells by treatment with CTLA-4 or IFN-γ (71). Decidual macrophages sorted for CD14^+^ have been reported to express IDO1 mRNA (72), although the purity of these cells was only 72–90%, so that it cannot be ruled out that contaminating cells rather than macrophages were responsible for the observed presence of IDO1 mRNA. Jones et al. implied the presence of IDO1 in mesenchymal stem cells grown from placentae, based on the observation that these cells suppressed allogeneic T cell proliferation in a manner partly dependent on IDO1 (73). Unpublished data show expression of IDO1 protein in stromal cells of the placental bed *post partum* (Astrid Blaschitz).

### TDO and IDO2

Limited information is available regarding the localization and role of TDO in the placenta. TDO mRNA and protein has been observed in mouse concept and placenta at a time preceding IDO1 expression (50). Dharane et al. reported TDO mRNA to be present in human placental explants (prepared following caesarian section) after 24 h of culture, and its expression increased following *ex vivo* exposure to LPS (66).

Indoleamine 2,3-dioxygenase-2 mRNA has been detected in term and, to a much lower extent, also in first trimester placentae (74). Isolated first trimester and term trophoblast cells as well as the BeWo choriocarcinoma cell line do not express IDO2 mRNA (74). Preliminary observations suggest, however, that both IDO2 and TDO protein are expressed in the human placenta (Astrid Blaschitz, unpublished data).

### Further enzymes involved in Trp degradation

Kynurenine 3-hydroxylase (KYN-OHase) catalyzes the oxidation of kynurenine to 3-hydroxykynurenine. KYN-OHase has been localized to glandular epithelial cells of first trimester decidua, as well as the syncytiotrophoblast, stroma, and macrophages of first trimester placenta. In term placenta, KYN-OHase expression was confined mainly to vascular endothelial cells of villous blood vessels, and to macrophages within the fetal villus (59). We are aware of only a single report of Tph (is it Tph-1?) in the cytoplasm of human cytotrophoblasts and syncytiotrophoblasts (75).

## Role of Trp Degradation

### General aspects

It has been known for decades that IDO1 is induced during infections and displays antimicrobial activity. Originally, induction of IDO has been observed in the lung following application of bacterial LPS (76) and infection with influenza virus (77). Such infection-associated induction of IDO1 was soon found to be mediated by IFN-γ (78). In a variety of different human cell lines, induction of IDO1 by IFN-γ is associated with growth inhibition of intracellular bacteria (such as *Chlamydia psittaci*) and protozoa (*Toxoplasma gondii*), as well an extracellular bacteria (14, 79, 80). In many though not all situations, addition of exogenous l-Trp attenuates growth inhibition, consistent with the notion that limitation of this essential amino acid by IDO1 at least in part explains the antimicrobial activity observed. The antimicrobial activity of IDO1 in human endothelial cells has been reviewed recently (81).

Oxidative degradation of Trp leads to both, a local depletion of Trp and formation of Trp metabolites. Both aspects are biologically relevant and have recently been reviewed (82), see also Table 2. For example, the Trp metabolites kynurenine (83) and kynurenic acid (84) are ligands of the AhR. Following ligand binding, this cytosolic transcription factor translocates into the nucleus where it binds to response elements in the promoters of target genes (85). In this way, kynurenine displays immunosuppressive properties by generating regulatory T (T_reg_) cells (86). The immunogenicity of DC is decreased, as AhR signaling induces DC to express IDO1 and IL10 (86–89). 3-Hydroxyanthranilic acid (3-HAA) as well as the other kynurenine metabolites anthranilic acid, quinolinic acid, and nicotinamide do not directly activate the AhR. Hydroxykynurenine does display an effect which, however, is weaker than kynurenine (86). On the other hand, 3-HAA has been suggested to prime DC for expressing reduced levels of pro-inflammatory cytokines, enhanced levels of TGF-β, and inducing T_reg_ cells (90, 91). The depletion of Trp also triggers amino-acid-sensing signal transduction pathways, such as the GCN2 kinase and inhibition of mTOR (92). The former pathway leads to cell-cycle arrest and functional anergy in CD8^+^ T cells (93). Lymphocytes are specifically affected by Trp depletion. This is because in these cells, IFN-γ does not induce tryptophanyl-tRNA synthetase so that lymphocytes are inefficient in competing for Trp compared with other cells (94, 95). In T helper cells, Trp depletion inhibits differentiation to Th_17_ cells (96) and it promotes *de novo* T_reg_ differentiation (97). IFN-γ is the main inducer of IDO in DC for the prevention of hyperinflammatory responses, whereas TGF-β confers regulatory effects on IDO independent of its enzymatic activity. In this case, IDO1 appears to act as a signaling molecule, by promoting complex formation of IDO1 with the tyrosine protein phosphatases SHP-1 and SHP-2. This leads to long-term tolerance via activation of SHP-1 phosphatase activity in plasmacytoid DC (98). Moreover, IDO1 plays an important role in the self-limitation of the immune response. Thus, short-term (4 h) activation of DC with IFN-γ and LPS leads to the induction of pro-inflammatory cytokines, while long-term (48 h) activation favors immunosuppression and tolerance via IDO1 signals (36, 82).

**Table 2:** **Pathways of immunomodulation by IDO1 and kynurenine pathway metabolites**.

Pathway	Functional consequence	Reference
IDO1 acting as a signaling molecule by complex formation with SHP-1 and SHP-2	Long-term tolerance in plasmacytoid DC	(98)
Depletion of Trp, activation of GCN2 kinase, and inhibition of mTOR in IDO-expressing cells	Cell-cycle arrest and functional anergy in CD8^+^ T cells	(92, 93)
Binding of kynurenine to AhR in DC and T cells	Decrease in immunogenicity of DC, and generation of T_reg_ cells	(86, 89)
3-HAA acting on DC (possibly via blocking the JNK and p38 MAPK pathways)	Decrease in expression of pro-inflammatory cytokines, increase in expression of TGF-β, and induction of T_reg_ cells	(90, 91)

As stated above, on one hand IDO1 generates metabolic products that induce T_reg_ cells, on the other hand T_reg_ cells can induce IDO1 expression (31). This suggests the presence of a positive feedback loop and raises the question of the limitation of this mutual interaction.

Indoleamine 2,3-dioxygenase-1-based suppression of immune reactions against foreign MHC-I molecules and minor histocompatibility antigens mediates feto-maternal tolerance (99, 100) also via induction of T_reg_ cells, which play a critical role in suppressing the anti-fetal immune response (101). The role for this in pregnancy has been questioned based on the fact that matings of allogeneic male and female IDO1^−/−^ mice yield viable offsprings (69). However, IDO2 and/or TDO may compensate for IDO1 and promote Trp metabolism in these mice, particularly as it is increasingly recognized that TDO expression is not limited to the liver. Rather, the enzyme is also present in mouse placenta (50).

Indoleamine 2,3-dioxygenase-1 mediates tolerance against tumors (102), and IDO inhibitors are being tested in clinical trials with patients suffering from cancer and chronic infections (103). Whereas IDO1 has been found in DC of tumor-draining lymph nodes (24), IDO1 could not be detected in regional lymph nodes of uteri of pregnant mice (P. Ack, Astrid Blaschitz, unpublished observations).

Trp metabolites also display non-immunological functions: for example, quinolinic acid and kynurenic acid have neuroactive properties (104–106), and 3-hydroxykynurenine and 3-hydroxyanthranilic acid display antioxidant activity (107). IDO1-mediated degradation of Trp in the endothelium of mice infected with malaria parasites or induced by endotoxemia contributes to the relaxation of arteries and to the control of blood pressure (63). Originally, kynurenine was reported to mediate arterial relaxation under these pro-inflammatory conditions, in part via activation of soluble guanylate cyclase. These findings were based on studies with commercial preparations of kynurenine (63). However, more recently, HPLC-purified kynurenine was found to be inactive, and IDO1-mediated vasorelaxation has been attributed to a yet to be identified Trp metabolite (Proceedings of the British Pharmacological Society at http://www.pa2online.org/abstract/abstract.jsp?abid\protect\kern+.1667em\relax=\protect\kern+.1667em\relax31322). Most recently, IDO1 has been reported to mediate angiotensin II-induced production of reactive oxygen species, apoptosis, and endothelial dysfunction (108).

The biological role of IDO2 is as yet unclear. Its Trp-degrading activity is much lower or even undetectable (15) compared with IDO1 (41), at least in the *in vitro* ascorbate/methylene blue assay commonly used (14). However, the probable physiological electron donor cytochrome *b*_5_ reduces recombinant mouse IDO2 and it increases its activity *in vitro* compared with that observed in the ascorbate/methylene blue assay (16). Human IDO2 expression is not able to rescue a yeast strain auxotrophic for nicotinic acid, suggesting it does not have sufficient activity to supply NAD^+^ in yeast (109). On the other hand, chemokine-induced production of kynurenine in human basal carcinoma cells correlated with the induction of mRNA expression of IDO2, but not IDO1 (110). It has been suggested that IDO2 activity is determined by the presence of particular co-factors that may be present only in certain cell types or conditions (40).

The high expression of TDO in the liver (111) makes it the key enzyme regulating circulating concentrations of l-Trp, and it is believed to have a major role in supplying NAD^+^ (112). TDO^−/−^ mice display increased plasma concentrations of Trp, leading to increased serotonin biosynthesis and alterations in behavior and neurogenesis (113). In analogy to IDO1, TDO activity also has been implicated in the inhibition of immune responses against tumors (56).

Hydroxylation by Tph-1 may also contribute to the exhaustion of Trp in a microenvironment, and it too has immunoregulatory effects. Tph-1 deficiency breaks allograft tolerance, induces tumor remission, and intensifies neuroinflammation. These effects are independent of the downstream product serotonin (114).

### Functional aspects of placental Trp catabolism

Localization of IDO1 in the utero-placental unit leaves us to speculate about its role at this site in particular: IDO1 in the epithelium of the mucosal surface and the glands of the endometrium and the decidua, and secretion of IDO [reflected in Trp-degrading activity in the cervical mucus (26)) may provide a mechanism of innate immunity against ascending infections of the female reproductive tract with intracellular bacteria such as *Chlamydia* but also against extracellular pathogens.

Endothelial cells may act as semi-professional antigen-presenting cells (115) and, as they degrade Trp, may contribute to the suppression of the immune response (31). Inhibition of IDO activity improves the ability of human umbilical vein endothelial cells to stimulate allogeneic T-cell responses. Transfection of these cells or human saphenous vein endothelial cells with the IDO1 gene, stimulates allogeneic T-cell responses and induces anergy in allospecific T cells (116). IDO1-positive endothelial cells of both the fetal and the maternal part of the placenta do not coexpress HLA-DR, which renders their contribution to the establishment and maintenance of feto-maternal tolerance unlikely. In situations where pro-inflammatory stimuli act on and induce MHC-II expression in placental endothelial cells, the ensuing immune response may, however, be modulated by endothelial IDO1. An antibacterial and antiparasitic role of endothelial IDO1 may be anticipated, and this might contribute to protection of the feto-placental unit against infection (81).

Endothelial catabolism of Trp by IDO1 in the villous chorion may also contribute to the regulation of the placental vasotonus. Preliminary data suggest that preconstriced human placental arterial rings relax in response to added Trp, and that this relaxation is partly inhibited by 1-MT (Roland Stocker, Peter Sedlmayr, unpublished observations). As the maintenance of placental perfusion is of crucial importance to the fetus, IDO1-induced relaxation of placental vessels may play an important role for feto-placental growth in the course of pregnancy. Moreover, on the other side of the interface, expression of IDO1 in the endothelium of spiral arteries may induce vasodilation and contribute to feeding blood into the intervillous space. This suggested role of IDO1 at this location might be a phenomenon particularly relevant after the first trimester of pregnancy, once the endovascular trophoblast plugs have vanished.

## Altered Trp Degradation in Pregnancy Pathology

There are reports of reduced placental IDO1 mRNA, protein, and placental Trp-degrading activity in preeclampsia, including a correlation between reduced placental Trp-degrading activity and the severity of the disease (27, 117–119). Not all studies, however, take into account that the gestational age of preeclamptic placentae needs to be matched to control placentae, as placental IDO1 expression normally increases with gestational age. Whereas the kynurenine-to-Trp ratio in plasma increases during normal pregnancy, in preeclampsia it remains unchanged and similar to that in non-pregnant women (117, 120).

In a model of pregnant mice carrying hemiallogeneic concept, pharmacological inhibition of IDO1 was reported to result in the mothers developing high blood pressure, proteinuria, and impairment of the local placental circulation, analogous to the lesions characteristic of human preeclampsia (121). In this model, 8-hydroxy-2′-deoxy-guanosine (8-OHdG, a marker for oxidative damage to DNA) was found to be higher in preeclamptic than normotensive pregnancies. Moreover, immunohistochemical signals of 8-OHdG inversely correlated with Trp-degrading activity, suggesting that a decrease in the antioxidant activity of IDO1 contributed to the pathogenesis of this disorder (122).

So far, little is known regarding the role of IDO1 in the context of intrauterine growth restriction (IUGR, synonymous with fetal growth restriction). There is one (however not in-depth) report stating that placentae in this disease show decreased IDO activity (123). Current interest focuses on a possible pathogenetic role of endothelial IDO1: in IUGR with and without preeclampsia chorionic vessels show reduced expression of IDO1, as assessed by immunohistochemistry, and a decrease in the relaxation of placental arteries induced *ex vivo* by added Trp (Roland Stocker and co-workers, unpublished).

Indoleamine 2,3-dioxygenase-1 expression in monocytes, macrophages, and DC of the decidua and of peripheral blood increases in normal pregnancy after treatment with CTLA-4 or IFN-γ whereas it decreases in spontaneous abortion (71). In allogeneic pregnancies in mice, application of 1-MT leads to T cell-mediated hemorrhagic necrosis and rejection of the conceptus soon after implantation (99, 100). This situation is similar to that of *in vivo* administration of an antibody against the T cell receptor β chain (124), and may be analogous to early pregnancy loss in humans, also called “chemical pregnancies.”

## Conclusion

Trp-degrading enzymes in the placenta lead to a deprivation of tryptophan and the formation of biologically active tryptophan metabolites at and near the sites of catabolism. The combination of these two processes has important consequences for the establishment and maintenance of feto-maternal immune tolerance. In addition, it may affect placental circulation and growth, as well as modulate local antimicrobial activity, the precise underlying mechanisms of which await elucidation. In particular, at present we lack detailed information on the expression, localization, and specific roles of IDO2 and TDO in the placenta. The occurrence of allogeneic pregnancies in IDO1^−/−^ mice suggests redundancy for the role of IDO1 in protecting against alloreactive maternal T cells, the mechanism of which needs to be uncovered. This might be done, e.g., by using various combinations of IDO1, IDO2, and TDO double gene knockout mice, perhaps in combination with pharmacological inhibition of the third Trp-oxidizing enzyme where appropriate.

## Conflict of Interest Statement

The authors declare that the research was conducted in the absence of any commercial or financial relationships that could be construed as a potential conflict of interest.
